# NADPH oxidase mediated oxidative stress signaling in FLT3-ITD acute myeloid leukemia

**DOI:** 10.1038/s41420-023-01528-5

**Published:** 2023-06-30

**Authors:** Yongfeng Chen, Zhenyou Zou, Mihnea-Alexandru Găman, Linglong Xu, Jing Li

**Affiliations:** 1 Department of Basic Medical Sciences, Medical College of Taizhou University, Taizhou, Zhejiang 318000 China; 2 Institute of Psychosis Prevention, Brain Hospital of Guangxi Zhuang Autonomous Region, Liuzhou, Guangxi 542005 China; 3 Faculty of Medicine, “Carol Davila” University of Medicine and Pharmacy, 050474 Bucharest, Romania; 4 Department of Hematology, Centre of Hematology and Bone Marrow Transplantation, Fundeni Clinical Institute, Bucharest, Romania; 5 Department of Hematology, Taizhou Central Hospital (Taizhou University Hospital), Taizhou, Zhejiang 318000 China; 6 Department of Histology and Embryology, North Sichuan Medical College, Nanchong, Sichuan 637000 China

**Keywords:** Oncogenesis, Mechanisms of disease

## Abstract

The internal tandem duplication of the juxtamembrane domain of the FMS-like tyrosine kinase 3 (FLT3-ITD) is the most common genetic change in acute myeloid leukemia (AML), and about 30% of all AMLs harbor a FLT3-ITD mutation. Even though FLT3 inhibitors have displayed encouraging effects in FLT3-ITD-mutated AML, the extent of the clinical response to these compounds is cut short due to the rapid development of drug resistance. Evidence has shown that FLT3-ITD triggered activation of oxidative stress signaling may exert a pivotal role in drug resistance. The downstream pathways of FLT3-ITD, including STAT5, PI3K/AKT, and RAS/MAPK, are considered to be major oxidative stress signaling pathways. These downstream pathways can inhibit apoptosis and promote proliferation and survival by regulating apoptosis-related genes and promoting the generation of reactive oxygen species (ROS) through NADPH oxidase (NOX) or other mechanisms. Appropriate levels of ROS may promote proliferation, but high levels of ROS can lead to oxidative damage to the DNA and increase genomic instability. In addition, post-translational modifications of FLT3-ITD and changes in its subcellular localization can affect downstream signaling which may also be one of the mechanisms leading to drug resistance. In this review, we summarized the research progress on NOX mediated oxidative stress signaling and its relationship with drug resistance in FLT3-ITD AML, and discuss the possible new targets in FLT3-ITD signal blocking to reverse drug resistance in FLT3-ITD-mutated AML.

## Facts

1. FLT3-ITD-triggered activation of oxidative stress signaling may play a critical role in drug resistance in AML.
2. Post-translational modification of FLT3-ITD and changes in its subcellular localization can affect downstream signaling which may be one of the mechanisms leading to drug resistance in AML.

## Open questions

1. What is the exact mechanism underlying the alteration of downstream signaling of the FLT3-ITD oncogene?
2. What is the exact relationship between post-translational modifications, subcellular localization, and FLT3-ITD oncogene signaling?
3. How to efficiently and persistently block FLT3-ITD oncogene signaling?

## Introduction

Abnormal signals from receptor tyrosine kinases (RTKs) are often discovered to exert a pivotal role in tumor initiation and progression. FLT3 protein belongs to the type III RTKs family and is solely expressed in normal bone marrow CD34^+^ stem/progenitor cells [1, 2]. The most frequent mutation that involves FLT3, which can occur in up to 30% of acute myeloid leukemia (AML) cases, is the internal tandem duplication (ITD) of its juxtamembrane domain [3]. The FLT3-ITD mutation causes constitutive activation of the tyrosine kinase domain, resulting in the autophosphorylation of the receptors and consequently in the phosphorylation of substrate proteins which in turn activate downstream pro-survival signaling pathways such as phosphatidylinositol 3‑kinase (PI3K)/protein kinase B (AKT), Ras/extracellular signal-regulated kinase (ERK) and Janus kinase (JAK)/signal transducer and activator of transcription (STAT), promoting the progression of leukemia [4–6]. Currently, it is well accepted that FLT3-ITD is an oncogenic driver mutations and a marker of poor prognosis in AML. According to a recent risk stratification study for AML patients, individuals with FLT3-ITD were less likely to achieve complete remission (OR) and were at increased risk of relapse-related mortality [7]. Accumulating evidences suggest that FLT3-ITD signal induced oxidative stress may involve in the pathogenesis of FLT3-ITD AML [8–10].

Several publications have delineated that the FLT3-ITD mutation is closely linked with an increased ROS production in AML cells [11, 12]. As a second signaling molecule, the role of ROS in FLT3-ITD AML is manifold. Moderate levels of ROS can act as pro-survival signals to promote cell proliferation. However, high concentrations of ROS can be responsible for oxidative damage and increase the probability of an erroneous repair of the DNA [13]. It is worth noting that quiescent AML LSCs generally have low ROS levels compared with cycling LSCs and bulk AML cells [14]. However, quiescent AML LSCs still inevitably suffer from oxidative DNA damage even in the absence of high ROS levels. Such damage increases genomic instability and drives the genetic evolution of LSCs, which is likely to give them competitive advantages [15]. Evidence of cytogenetic evolution has shown that the rapid clonal evolution through genomic instability may be the main reason for the high relapse rate and chemorefractoriness in AML [16]. In a recent study, Schmalbrock et al. investigated the clonal evolution patterns of FLT3 ITD AML patients receiving Midostaurin treatment to explore the potential mechanisms for tyrosine kinase inhibitors (TKI) resistance. The results showed that more than half of the patients acquired new mutations at the time of disease resistance or progression. However, in 32% of cases, no FLT3-ITD mutational change was observed, suggesting there are other resistance mechanisms which can bypass FLT3 inhibition [6].

Recently, numerous studies have shown that the post-translational modifications that involve FLT3-ITD and its localization in cells can lead to changes in downstream signaling which may also be another mechanism leading to TKI drug resistance. Therefore, effective targeting of FLT3-ITD in the personalized approach to AML management necessitates an in-depth understanding of the underlying links between FLT3-ITD localization, activity, and downstream signaling.

## FLT3-ITD subcellular localization

The FLT3 gene is located on chromosome 13q12 in humans. This gene encodes a protein consisting of 993 amino acids, whose structure is made up by an “N-terminal extracellular domain, a transmembrane region, a juxta-membrane domain, and a C-terminal cytoplasmic tyrosine kinase domain” [4]. The FLT3 protein is co-translationally translocated into the endoplasmic reticulum (ER) and is then subjected to a multistep glycosylation process and folding at the ER. The control system in the ER guarantees that solely correctly folded FLT3 molecules leave the ER, undergo further glycosylation in the Golgi apparatus, and ultimately move into the plasma membrane (PM) [17].

Schmidt-Arras et al. highlighted the existence of two forms of the FLT3 receptor *via* western blotting analysis. One 150 kDa receptor represents a complex glycosylated mature form, and the other 130 kDa receptor represents an underglycosylated immature form with a mannose-rich structure. The 130-kDa forms were predominantly detected in cells expressing FLT3-ITD, whereas 150 kDa forms were found to have a higher proportion in the cells expressing FLT3-WT [18]. Multiple studies have shown that activated mutations lead to altered subcellular localization of the FLT3 protein. Several investigations have demonstrated that the FLT3 wild-type protein is mainly located at the PM, while the FLT3-ITD protein is mainly detected in the ER, Golgi apparatus, and perinuclear region, but rarely or even unable to be detected in the PM [19, 20]. Evidence suggests that an altered localization of FLT3-ITD may affect its downstream signaling and may be associated with the development of drug resistance [20–22].

Currently, the mechanism by which activated mutations lead to altered FLT3-ITD subcellular localization is unclear. Besides being possibly related to the glycosylation status, recent studies have found that palmitoylation and phosphorylation may be two other key factors regulating FLT3-ITD intracellular localization. According to Lv et al., the S-palmitoylation of FLT3-ITD is responsible for the inhibition of FLT3-ITD’s surface expression, and the application of the palmitoylation inhibitor 2-bromopalmitate (2-BP) can decrease the ER retention of FLT3-ITD and promote its transportation to Golgi and cell surfaces [19]. Similarly, the application of tyrosine kinase inhibitors (TKIs) can also increase FLT3-ITD’s plasma membrane expression levels, which may be due to the fact that TKIs can inhibit the phospho-dependent interaction of FLT3-ITD with ER resident proteins and promote FLT3-ITD glycosylation [18, 19, 23]. Elevated FLT3-ITD plasma membrane expression levels following the use of TKIs have been reported to enhance the efficacy of chimeric antigen receptor T-cell therapy and antigen-dependent cytotoxicity based on anti-FLT3 compounds [23], suggesting that combining TKIs with immunotherapy may help improve prognosis for AML patients.

## NOX play a key role in FLT3/ITD signaling

AML is a group of hematologic malignancies with high heterogeneity which is also extended to their metabolic profiles [24–26]. It was reported recently that FLT3/ITD induce a significant increase in aerobic glycolysis via AKT-mediated upregulation of mitochondrial hexokinase (HK2), and render AML cells highly dependent on glycolysis [25]. However, another study of Erdem et al. showed that compared to FLT3wt AML cells, which are reliant on glycolysis, FLT3-ITD AML cells are more OXPHOS-driven [26]. Besides, studies also showed that AML LSCs seem to mainly rely on mitochondrial respiration, therefore, may actually be particularly sensitive to OXPHOS inhibition [14, 26].

ROS are an important by-product of aerobic metabolism. Excessive production of ROS is frequently observed in FLT3-ITD AML and is demonstrated closely associated with the pro-survival mechanism of AML cells [27–29]. In eukaryotic cells, ROS can be produced by a number of approaches, including mitochondria, the xanthineoxidase system, and the NOX system, etc. [13]. However, it was reported that in AML cells, the increase of ROS is mainly attributable to the constitutive activation of NOX. In contrast, overproduction of mitochondrial ROS was rarely observed [27–29]. NOX is a multienzyme complex that uses cytoplasmic NADPH as electron donor to catalyze the production of the superoxide anion (O_2_^−^) from extracellular O_2_ [30]. NOX includes 7 isoforms, i.e., dual oxidase (DUOX) 1–2 and NOX1-5. Each isoform displays different mechanisms of regulation. The first four isoforms (NOX1-4) all require binding to p22Phox (a membrane bound NOX complex subunit) to secure an accurate post-translational modification, long-term stability and membrane targeting. Furthermore, with the exception of NOX4, all these proteins require additional regulatory subunits for their activity, which have to translocate from the cytosol to form a functional complex with the membrane-bound NOX [30, 31].

Among all NOX isoforms, NOX1, NOX2 and NOX4 are expressed by human CD34^+^ hematopoietic progenitor cells [30–32]. Among them, NOX2 and NOX4 are critical players in the generation of ROS via FLT3-ITD signaling [11, 33]. Sallmyr el al. found that FLT3-ITD signaling promotes binding of Rac1 GTPase to phosphorylated STAT5 (pSTAT5) in FLT3-ITD-transfected and positive cells, which in turn enhances NOX2 activity and promotes ROS production. Application of FLT3 inhibitors blocked ROS generation, resulting in reduced double-stranded DNA breaks (DSBs), and enhanced efficiency and fidelity of the DNA repair processes [8]. Likewise, there is evidence that FLT3-ITD signaling promotes NOX4 expression and the generation of ROS *via* STAT5 mediation. Furthermore, it facilitates the inactivation of one of its negative regulators, that is, the protein-tyrosine phosphatase density-enhanced phosphatase-1 (DEP-1), by reversible oxidation of its catalytic cysteine residue. NOX4 knockdown reduces ROS concentrations, restores the activity of DEP-1, and reduces FLT3-ITD-driven transformation [33].

Recently, Moloney et al. reported that PM-located FLT3-ITD can promote NOX4 activation by activating AKT, thereby inducing the production of pro-survival ROS. This process depends on the AKT activation and only PM- located FLT3-ITD can activate AKT. Therefore, it is believed that only FLT3-ITD at the PM is carcinogenic, and targeting PM-localized FLT3-ITD may prove beneficial in the prevention of downstream ROS-driven carcinogenesis [11]. In another study, Lv et al. used 2-BP to inhibit the palmitoylation of FLT3-ITD and discovered that the enhanced expression of FLT3-ITD in the PM can promote oncogene signaling mediated by AKT and other pathways, thereby promoting the proliferation of leukemia cells [19]. The result of the study by Lv et al. confirmed that the PM-located FLT3-ITD is the main source of oncogene signaling. However, there is evidence indicating that oncogene signaling is originated not only from the PM-located FLT3-ITD, but also from the FLT3-ITD located in early secretory compartments [20]. Currently, the subcellular localization of FLT3-ITD and its impact on downstream signaling pathways has become a hot topic in AML research, an in-depth understanding of the FLT3-ITD signal and its downstream pathways will help to precisely suppress the transmission of AML oncogene signals.

## Impact of subcellular localization of FLT3-ITD on downstream signaling pathway

The downstream signaling pathways of the FLT3-ITD oncogene, e.g., PI3K/AKT, JAK/STAT, and Ras/ERK, are representative signaling pathways that are abnormally activated in AML. The aberrant FLT3-ITD oncogene signal constitutively activates the above signaling pathways, which subsequently inhibit cell apoptosis, promote cell proliferation and survival *via* regulation of apoptosis-related genes, thereby leading to the initiation and development of AML [34–37].

**Fig. 1:**
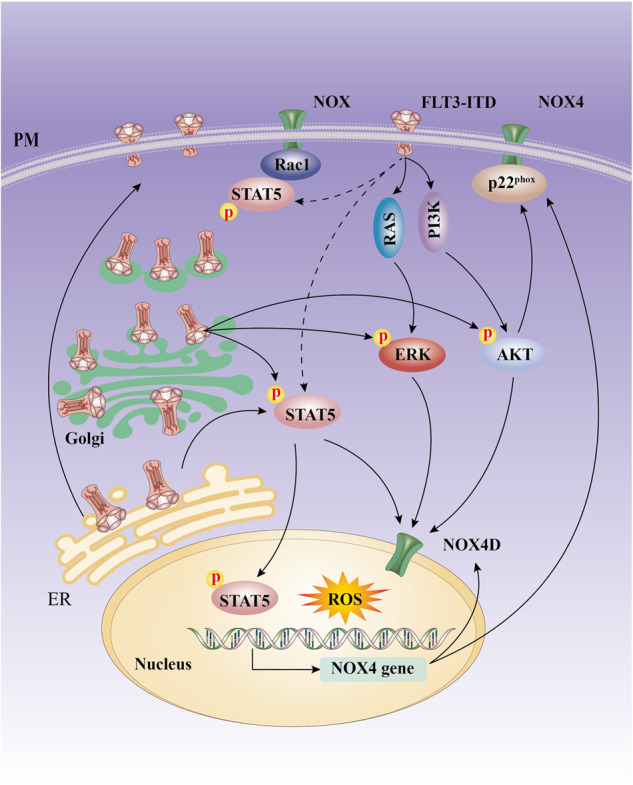
NOX mediated oxidative stress signaling in FLT3-ITD AML. The subcellular localization of FLT3-ITD may affect its downstream signaling (see main text, the dotted lines indicate a relatively weak effect) [20, 21]; The PI3K/AKT, STAT5, and ERK1/2 downstream pathways of FLT3-ITD can promote ROS generation through NOX4D localized at the nuclear membrane [11, 40]; FLT3-ITD signaling causes STAT5 phosphorylation, P-STAT5 subsequently translocates to the nucleus and activates the transcription of NOX4; P-STAT5 can also maintain the active form of RAC1, and promote RAC1 combination with NOX, leading to an increase in ROS generation; FLT3-ITD signaling also stabilizes p22^phox^ protein *via* AKT pathway and increases ROS generation [93]. The dotted line in the figure indicates a relatively weak effect. This figure was drawn based on existing research data; its accuracy and more precise signaling mechanisms must be confirmed and supplemented by extensive, in-depth studies.

Recently, several studies have highlighted that the intracellular disposition of FLT3-ITD may determine which downstream pro-survival signals are activated constitutively by FLT3-ITD. In particular, the PM-localized FLT3-ITD strongly activates the mitogen-activated protein kinase (MAPK) and PI3K pathways with diminished STAT5 phosphorylation [20]. Golgi-localized FLT3-ITD activates STAT5, AKT and ERK [19, 21], whereas ER-retained FLT3-ITD aberrantly activates STAT5 and upregulates its targets, Pim-1/2, but fails to activate AKT and ERK signaling [21, 22] (Fig. 1). Currently, it is believed that resistance to TKIs can also develop as an “off-target” mechanism when changes occur in the subcellular localization of FLT3-ITD and result in alterations of downstream pathways [20–22]. In the study of Fleischmann et al., pre-treatment with N-glycosylation inhibitors (tunicamycin or 2-deoxy-D-glucose) led to intracellular retention of FLT3-ITD, and the susceptibility of FLT3-ITD-expressing AML cells to 17-AAG (HSP90 inhibitor) was increased. 17-AAG treatment induced degradation of FLT3-ITD and promoted apoptosis in AML cells. In contrast, histone deacetylase inhibitor valproic acid (VPA) pre-treatment significant increased the surface expression of FLT3-ITD, phosphorylated ERK and phosphorylated AKT were then increased, and the susceptibility of AML cells to rapamycin was enhanced [22]. The result of the study by Fleischmann et al. indicates that, the allocation of FLT3-ITD to different cellular compartments and targeting distinct downstream signaling might represent a new therapeutic strategy to overcome resistance towards tyrosine kinase inhibitors in FLT3-ITD AML.

Ras/ERK, PI3K/AKT and JAK/STAT are the main oxidative stress signaling pathways and can induce pro-survival ROS production through different mechanisms, such as inhibiting the activity of forkhead O box (FOXO) or enhancing NOX expression [38, 39]. Recently, Moloney et al. found that in AML cells, the PI3K/AKT, STAT5, and ERK1/2 downstream pathways of FLT3-ITD can promote ROS generation through NOX4D (NOX4 28 kDa splice variant) localized at the nuclear membrane, and inhibiting these pathways could lead to a reduction in ROS production [40], suggesting that NOX4D localized at the nuclear membrane is the main source of pro-survival ROS production and is also a key factor leading to genomic instability and DNA damage [40].

Mounting evidence have shown that, secondary mutations caused by accumulation of DNA damage may critically contribute to the development of drug resistance [41–45]. However, the precise role of secondary mutations in relapse and TKIs resistance still requires further study. Recent several studies have showed that FLT3-ITD-induced ROS generation can result in the oxidation of tumor suppressor genes and of DEP-1 phosphatase which acts as negative regulator of FLT3. Moreover, mutagenesis can directly derive from DNA oxidation if DEP-1 inactivation is not repaired correctly [33, 46, 47]. Genomic instability mediated by ROS in FLT3-ITD AML progression has been excellently reviewed by Rebechi et al. [16].

## Redox control is crucial to the survival of AML

ROS represent a group of oxygen-containing and highly reactive molecules which serve as important second messengers involved in a variety of signal transduction that regulates cell growth, proliferation, and differentiation of cells [13]. It is now accepted that the appropriate concentration of ROS is crucial for cancer cell survival, however, excessive ROS concentrations may cause both severe oxidative damage and eventually cell death [13]. Therefore, the maintenance of redox balance is crucial for any cell [48, 49].

**Fig. 2:**
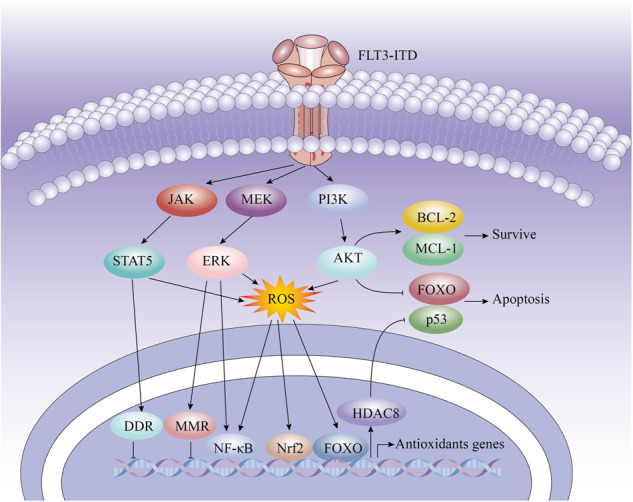
Redox balance and survival maintenance signaling in FLT3-ITD AML. FLT3-ITD signaling can upregulate the expression of key DDR factors and MMR factors through the STAT5 and ERK pathways, respectively [10]; The nuclear translocation of redox sensitivity transcription factors, such as Nrf2, NF-κB, and FOXO, etc. can be promoted by ROS, which promote the transcriptional activation of antioxidant defense genes and enhance the antioxidant capacity of cells [28, 51–53]; FOXO can promote the transcription of HDAC8, and then inhibit the antitumor activity of p53 *via* deacetylation [94]; FOXO also has pro-apoptotic effect, but this effect is inhibited by AKT, whereas the pro-survival effect of BCL-2 and MCL-1 is activated [35, 58–60]. This figure was drawn based on existing research data; its accuracy and more precise signaling mechanisms must be confirmed and supplemented by extensive, in-depth studies.

Like other cell types, leukemia cells also have well-established and complex mechanisms for maintaining their redox balance, and the level of pro-survival ROS is finely regulated in AML. It was reported that in FLT3-ITD AML, elevated ROS concentrations induced by oncogene signaling lead to compensatory enhancement of antioxidant capacity that protecting leukemia cells from oxidative damage, thereby bringing leukemia cells survival benefits [8, 50]. An example of an oxidative stress signaling-triggered mechanism of protection is regulating nuclear translocation of redox sensitivity transcription factors, e.g., FOXO, nuclear factor (erythroid-derived)-like 2 (Nrf2), hypoxia inducible factors (HIFs), Kelch-like ECH-associated protein 1 (Keap1) etc. Activation of these transcription factors can promote the transcriptional activation of antioxidant defense genes and enhance the cells’ antioxidant capacity to avoid overproduction of ROS [28, 51–53] (Table 1). Interestingly, FOXO can set off the antioxidant system to promote the survival of AML cells and also enhance apoptosis. However, in FLT3-ITD AML cells, the pro-apoptotic effect of FOXO is inhibited by Akt [54] (Fig. 2).

**Table 1:** The main ROS-dependent transcription factors and antioxidant proteins in AML.

Transcription factor/Antioxidant protein	Function	Ref.
ATM	Promoting antioxidant response through activation of glucose-6-phosphate dehydrogenase (G6PD)	[77]
FOXOs	Inhibiting ROS generation by upregulating the transcription of antioxidant genes (e.g., ATM) and antioxidant enzymes (e.g., SOD and CAT)	[78]
HIFs	Protecting AML cells *via* inhibiting the generation of ROS	[79]
HO-1	Under oxidative stress, HO-1 expression is induced by the NRF2 transcription factor as part of the cellular antioxidant defense response	[29]
NF-κB	Regulator of genes involved in regulating the ROS level in cells (e.g., Nrf2)	[38, 80]
Nrf2	Mediating cytoprotective antioxidant responses *via* upregulating the transcription of antioxidant genes (e.g., HO-1)	[8, 81]
Prdx	Protecting intracellular genomic DNA, lipids and proteins from oxidative damage by regulating intracellular ROS levels	[82]
Trx	Maintaining intracellular redox homeostasis, reducing oxidized protein levels and protein-refolding activity to limit damage caused by oxidative stress	[83]

*ATM* Ataxia telangiectasia mutated, *FOXO* Forkhead box O, *HIF* hypoxiainducible factor, *HO-1* Heme oxygenase-1, *NF-κB* nuclear factor-kappa, *Nrf2* NF-E2-related factor 2, *Prdx* peroxiredoxin, *Trx* thioredoxin.

A commonly accepted chemoresistance mechanism is the oncogenic kinases-driven DNA damage repair activity enhancement which favors the survival of malignant cells under genotoxic stress [55]. Recently, Wu et al. reported that in FLT3-ITD-mutated AML, FLT3-ITD signaling can upregulate the expression of key DNA damage response (DDR) factors, e.g, Wee1-like protein kinase (WEE1), checkpoint kinase 1 (CHK1), proviral integration site for Moloney murine leukemia virus-1 (PIM-1), radiation sensitive 51 (RAD51), and mismatch repair (MMR) factors, e.g, MutS homolog 2 (MSH2), MSH6 and MutL homolog 1 (MLH1) through the STAT5 and ERK pathways, respectively (Fig. 2). These responses could promote AML cell survival under oxidative stress stimulation and induce chemoresistance [10]. In a FLT3-ITD AML mouse model, animals treated with the MK8776 (CHK1 inhibitor) and cytarabine survived longer than those treated with cytarabine alone, suggesting that the combination of DNA damage response inhibitors and conventional chemotherapy may be useful in the treatment of FLT3-ITD AML [10].

Previous studies have shown that suppression of apoptosis signals can also help AML cells maintain survival under oxidative stress. Hole et al. reported that AML cells can escape the oxidative damage by inhibiting p38^MAPK^-mediated cycle arrest and/or apoptotic signaling [27]. Besides, it was found that the expression or activity of pro-apoptotic proteins, e.g., B-cell lymphoma 2 (Bcl-2) antiagonist of cell death (Bad) and Bcl-2-associated X protein (Bax) are inhibited in FLT3-ITD AML cells [56, 57], whereas the expression or activity of pro-survival proteins, e.g., B-cell lymphoma extra large (Bcl-xL), Bcl-2 or myeloid cell leukemia-1 (MCL-1) are upregulated, which is conducive to AML cells’ survival [35, 58–60]. Therefore, inhibiting the antioxidant capacity of leukemia cells, amplifying oxidative stress signaling, and restoring/activating apoptotic pathways may help to eradicate AML cells [61–65].

## Blockade of FLT3-ITD signaling

As the FLT3-ITD oncogene signal is a crucial player in the development of AML, it has emerged as a relevant target in the management of this malignancy. To date, several FLT3 inhibitors have been developed, tested in randomized clinical trials, i.e., midostaurin, gilteritinib, quizartinib, and other TKIs, and approved for clinical application. On this basis, the development of TKIs with multi-target inhibition has been continuously strengthened to improve their efficiency and durability on mutant FLT3 kinases (Table 2). Furthermore, the search for new therapeutic targets is also ongoing. Recent studies have demonstrated that activating mutations in FLT3 can induce an unstable conformation of the kinase, leading to its high dependence on chaperones, e.g., heat shock protein (HSP) 90. Therefore, FLT3-ITD is highly sensitive to the action of HSP90 inhibitors, and treatment with HSP90 inhibitors such as 17-AAG and LAM-003 could result in FLT3-ITD degradation [22, 66]. As NOX is the major source of ROS in FLT3-ITD AML, it is also an ideal target to inhibit FLT3-ITD pro-survival signaling. In FLT3-ITD AML cells, NOX2 inhibitors have been reported to cause decreased intracellular concentrations of ROS, inhibiting FLT3’s growth and survival downstream pathways, and increasing apoptosis associated with restoration of p38^MAPK^ and induction of mitochondrial ROS [67]. Downregulation of NOX4 expression by RNA interference or treatment with NOX4 inhibitors such as schisandrin B also notably decreased ROS production in FLT3-ITD-mutated AML, and reduced both in vitro and in vivo FLT3-ITD-driven signaling and cell transformation [33].

**Table 2:** Novel TKIs in the study of FLT3-ITD AML treatment.

Drug	Description	Function	Ref.
AMG 925	A potent, selective, and bioavailable FLT3/CDK4 dual kinase inhibitor	Inhibits FLT3 mutants (e.g., D835Y) that are resistant to the current FLT3 inhibitors (e.g., AC220 and sorafenib)	[84]
Cabozantinib	A multi-targeted TKI of FLT3, MET, AXL, VEGFR, and KIT	Inhibits FLT3-ITD tyrosine kinase in a potent and sustained fashion	[85]
EC-70124	A hybrid indolocarbazole analog with a potent and selective inhibitory effect on FLT3	Potently inhibits wild-type and mutant FLT3, and also other important kinases such as PIM kinases	[86]
FN-1501	A potent inhibitor of FLT3 and other tyrosine kinases such as CDK4/6, KIT, PDGFR, ALK and RET tyrosine kinase proteins	Antiproliferative activities against FLT3-ITD expressing cell line MV4-11	[87]
HM43239	A active small molecule inhibitor of FLT3-ITD, FLT3-TKD, and FLT3- ITD/TKD double mutations	Overcomes the FL-induced drug resistance with a higher cytotoxic potency in MOLM-14 cells harboring FLT3-ITD	[88]
MZH29	A type II FLT3 inhibitor that tolerated the F691L mutation	Showed sustained inhibitory effects on wild-type and mutant FLT3, including the FLT3-ITD, FLT3-D835H/Y/V and FLT3-K663Q mutants	[89]
Pexidartinib	A small molecule TKI with selective activity against the CSF1 receptor, KIT and FLT3-ITD	Having inhibitory activity against the FLT3 TKI-resistant F691L gatekeeper mutation in relapsed/refractory FLT3-ITD-mutant AML	[90]
SEL24	A dual PIM and FLT3-ITD inhibitor	Significant inhibitory activity on FLT3-ITD and tyrosine kinase domain (TKD) positive AML	[91]
SKLB-677	An FLT3 and Wnt/β-catenin signaling inhibitor	Showed considerable suppression effects on leukemia stem-like cells in in vitro functional assays, but had no influence on normal HSCs	[92]

*ALK* anaplastic lymphoma kinase, *CDK* cyclin-dependent kinase, *CSF1* colony-stimulating factor 1, *KIT* KIT proto-oncogene receptor tyrosine kinase, *PDGFR* platelet-derived growth factor receptor, *R/R* relapsed/refractory, *RET* rearranged during transfection, *Wnt*, β-catenin.

Protein post-translational modifications (PTMs) are important in the regulation of protein function. As mentioned above, PTMs such as glycosylation and palmitoylation not only affect the subcellular localization of the FLT3-ITD protein, but also lead to alterations in downstream pathways, which may further lead to drug resistance. Therefore, targeting the PTMs of FLT3-ITD protein to overcome drug resistance of AML cells has become a hot topic of current research. According to Williams et al., a lipophilic statin, i.e., fluvastatin, can decrease the mutant FLT3’s kinase activity *via* preventing advanced glycosylation of the receptor, reducing FLT3-ITD cell surface expression, inhibiting the activation of MAPK and AKT, and reducing the TKI drug resistances while leading to the apoptosis of FLT3-ITD-mutated AML cells [68]. The antineoplastic pharmacological agents 2-deoxy-d-glucose (2-DG) [69] and tunicamycin [70] have also been found to exert pro-apoptotic actions *via* glycosylation inhibition, as well as FLT3-ITD cell surface expression and signaling. Moreover, the co-administration of FLT3-ITD inhibitors and tunicamycin enhanced cell death synergistically, in particular in FLT3-ITD-mutated AML cells [70]. Recently, an investigation conducted by Lv et al. discovered that palmostatin B, a pan-depalmitoylase inhibitor, dose-dependently inhibited FLT3-ITD cell surface expression, as well as AKT/ERK activation in FLT3-ITD-mutated AML, thereby inhibiting cell growth. Moreover, survival of FLT3-ITD-mutated AML cells was suppressed by the synergism between gilteritinib, a FLT3 kinase inhibitor, and depalmitoylation inhibitors. Thus, depalmitoylation may emerge as a future therapeutic target in FLT3-ITD-mutated AML [19].

Blockage of intracellular transport routes of FLT3-ITD has also been shown to effectively inhibit oncogenic signals. Yamawaki et al. reported that the use of 2-methylcoprophilinamide (M-COPA) or brefeldin A to inhibit FLT3-ITD’s export from the ER or the use of monensin to inhibit FLT3-ITD’s export from the Golgi apparatus can notably decrease signals of tyrosine phosphorylation in AML [21]. In addition, according to Moloney et al., retention of FLT3-ITD in the ER can occur following treatment with brefeldin A and tunicamycin, leading to reduced NOX4 and p22phox – the partner protein of NOX4—expression. Consequently, the generation of pro-survival ROS is blocked [40]. As FLT3-ITD is a misfolded protein, it is noteworthy to mention that the massive accumulation in the ER of misfolded proteins leads to increased cellular susceptibility to ER stress and oxidative stress [71]. As reported by Masciarelli et al., a combination therapy consisting of low concentrations of retinoic acid, tunicamycin, and arsenic trioxide can induce oxidative stress and ER stress, resulting in FLT3-ITD-mutated AML cells’ apoptosis. Based on the aforementioned findings, it seems that AML cells are sensitive to ER stress and oxidative stress which may lay the path for novel therapeutic targets to eradicate this hematological malignancy [71].

The strategy of blocking the downstream signal of FLT3-ITD has also attracted much attention. For example, the oncogenic serine/threonine kinase Pim-1 is transcriptionally upregulated downstream of FLT3-ITD and stabilizes and phosphorylates FLT3-ITD, thus promoting the signaling of FLT3-ITD in a positive feedback loop in AML cells [72]. Baldwin et al. explored the synergism between quizartinib (FLT3 inhibitor) and azd1208 (PIM inhibitor) in FLT3-ITD-mutated AML. As compared to their singular use, the combined use of these two pharmacological agents showed synergistic effect and more efficiency in inhibiting AML cells’ growth and enhanced their apoptosis both in vivo and in vitro. The explanation behind this result could be that this drug combination downregulates the anti-apoptotic protein Mcl-1’s expression [72]. According to Jasek Gajda et al., another combination of pharmacological agents, namely ZSTK474 (PI3K inhibitor) and AZD0364 (ERK1/2 inhibitor) can suppress the in vitro activation of Akt and ERK1/2 in molm-14 cells, i.e., in a cellular model of FLT3-ITD-mutated AML, induce the apoptosis of AML cells by promoting the generation of ROS [48]. In vivo and in vitro experiments have confirmed that AC-4-130, an inhibitor of the SH2-domain of STAT5, is able to suppress the pathological activity of STAT5 in FLT3-ITD-mutated AML, thereby effectively inhibiting the proliferation of AML cells [73].

## Conclusions and future perspectives

The FLT3-ITD mutation is one of the most important gene mutations in AML, and it is also a crucial indicator of poor prognosis in this blood cancer. Current second-generation TKIs, such as sorafenib, gilteritinib, crenolanib, and quizartinib, are highly specific and potent, and often lead to favorable outcomes when employed in the early treatment of FLT3-ITD-mutated AML. However, the efficacy of TKIs is often challenged by the development of resistance caused by a myriad of factors, e.g., secondary mutations or signaling compensations. Therefore, most of these drugs fail to achieve the expected efficacy.

Recently, a global untargeted metabolomics and stable isotope-labeling mass spectrometry analysis was applied to evaluate sorafenib resistance in FLT3-ITD-mutated leukemic cells [50]. It was shown that resistant cells display fundamentally rewired metabolic profiles, characterized by elevated need for glucose, accompanied by a reduction in glucose flux into the pentose phosphate pathway. Besides, increased oxidative stress was accompanied by an enhanced antioxidant capacity of glutathione. These results demonstrate that FLT3-ITD-mutated leukemia cells have obvious metabolic and redox adaptability which may contribute to the development of sorafenib resistance [50]. Based on transcriptomic analyses, Zavorka Thomas et al. identified glutamine transporter SNAT1 (SLC38A1) as a novel target for gilteritinib. In addition, it was highlighted that impaired glutamine uptake and utilization within leukemic cells can enhance the efficacy of gilteritinib [74]. Similarly, another FLT3 inhibitor, namely AC220, causes impaired glutamine metabolism in AML, particularly when co-administered with CB-839, i.e., an inhibitor of glutaminase, leading to elevated ROS production in the mitochondria of leukemic cells and depleting their glutathione sources. Thus, this treatment promotes cell death in this blood cancer, suggesting new therapeutical strategies for a subset of relapsed/refractory AML [75].

The use of antioxidants during leukemia therapy is also currently a hot topic. It was recently reported that N-acetyl-cysteine, a natural product, was able to reduce ROS generation in FLT3-ITD-mutated AML and contributed to the prevention of aberrant interchromosomal homologous recombinations which are involved in leukemia survival and progression [76]. Theoretically, the use of antioxidants may help reduce TKI resistance caused by secondary mutations. However, antioxidants will inevitably weaken the therapeutic effect of pro-oxidant chemotherapy drugs. Therefore, exploiting new antioxidants with synergistic anti-leukemia effects may aid in providing solutions to this unsolved dilemma.
